# Orthodontics social media, perceptions of science- and non-science-based posts among orthodontists, dentists, students and laypeople

**DOI:** 10.1371/journal.pone.0286927

**Published:** 2023-09-29

**Authors:** Gil Guilherme Gasparello, Sergio Luiz Mota-Júnior, Giovani Ceron Hartmann, Augusto Hideki Berlesi, Fábio Acciaris, Letícia Machado Berretta, Matheus Melo Pithon, Orlando Tanaka

**Affiliations:** 1 Orthodontics, Medicine and Life Science School, Pontifícia Universidade Católica do Paraná, Curitiba, Brazil; 2 Department of Orthodontics, Juiz de Fora Federal University, Juiz de Fora, Minas Gerais, Brazil; 3 Private Practice, Santos, Brazil; 4 Department of Orthodontics, Southwest Bahia State University—UESB, Jequié, Bahia, Brazil; Hamadan University of Medical Sciences School of Dentistry, ISLAMIC REPUBLIC OF IRAN

## Abstract

Worldwide, social media is gaining popularity year after year. In Brazil, by 2027, there will be more than 188 million users of social media sites, against 165 million in 2022, therefore, the usage of general population and health care professionals, including orthodontists, is increasing. Differently from scientific journals that undergo a rigorous peer review process prior to publication, the same level of demand is not found on social media. Hence, this study aimed to assess whether orthodontists can recognize scientifically based and non-science-based posts and if their perceptions are different from general opinion (laypeople), dentistry students, and dentists (non-orthodontists). The posts were created using the search tool on Instagram with the hashtags #clearaligners #acceledent #selfligatingbraces and #propelorthodontics, four scientific based posts and four without or with low scientific evidence were selected and evaluated through a virtual questionnaire in the QUALTRICS platform by 385 people, (175 laypeople, 102 dentists, 58 dentistry students, and 50 orthodontists) using a visual analogue scale (VAS). In addition, four questions were asked. ANOVA (Adjusted Bonferroni correction) and Pearson’s chi-squared, and Student T tests were applied to identify statistical differences. Significant difference was found only for posts with no scientific evidence between orthodontist’s group when comparing with laypeople (*p* < 0.001) for the #selfligatingbraces, and the #propelorthodontics for the group orthodontists when compared with laypeople (*p* = 0.031) and dentists (*p* = 0.033). Instagram was the favorite social media where most of the participants spend more than 3 h. Of the participants, 97% used social media to keep informed and read the news. Almost half of the respondents search for orthodontics services on social media. Orthodontists were able to perceive differences in only two posts from the general perception (laypeople) regarding non- or low-scientific-evidence posts.

## Introduction

Currently, social media has become the largest means of direct or indirect communication, with more than 3.5 billion daily users worldwide who spend an average of 3 h a day online [1]. One of the current concerns of health professionals is how they can effectively transmit information to patients so that their knowledge about various aspects of health is reinforced. In 2015, 89% of orthodontic patients and parents reported using social media [2] In this context, social media can be considered a key tool to be considered by dentists in an attempt to bring patients to their offices [3].

Online social media had an initial focus on large-scale, unidirectional dissemination of information using portals, websites, and personal pages. In addition to being a means to establish interpersonal relationships, online social networks have become interactive platforms that offer a large variety of additional services [4].

Most patients using social media reported it helps in collecting information about orthodontic treatment [5]. The main reasons patients reported searching on social media were to obtain information about orthodontic treatment outcomes, types of appliances, and data about practical aspects of treatment [6]. However, orthodontic advertisements are recognized as lacking a robust evidence base, and of course misinformation and false information can be dangerous [7, 8]. Marketing claims related to orthodontic products published in scientific journals were, for example, based on a high level of evidence in less than 2% of cases [9].

Concerns were expressed that although articles submitted to scientific journals are often subject to a rigorous peer review process before publication, the same standards are not applied to social media posting. Although some studies report posts lacking scientific evidence on social media [9–11] it is unclear whether the readers know or believe everything they see and read through social media screens.

Hence, this study aims to assess whether orthodontists can recognize scientifically based and non-science-based posts and if their perceptions differ from general opinion (laypeople) as well as dentistry students and general dentists (non-orthodontists).

## Methods

This cross-sectional study was held in Brazil and approved by the Pontifícia Universidade Católica do Paraná ethics committee under number 2,235,302. All patients provided informed consent to participate in this research study at the moment they agreed to complete the online survey. The objective study population consisted of anyone over the age of 18, complete and respond all questions of questionnaire. Laypeople, dentistry students, general dentists, and orthodontists from Brazil were included.

A sample calculation was performed to define the number of individuals targeted to participate in the research. A sample number of 385 people was determined based on the Brazilian population, considered as an infinite population, a 95% confidence level, and a 5% error margin, in which Z = 1.96 corresponding to the abscissa of the standardized normal curve, for a 95% confidence level, e = margin of error considered 0.05 and *p* the standard deviation of 0.5.

### Posts and questionnaire construction

To create the posts to be evaluated, the search tool on Instagram was used separately by three researchers with the hashtags #clearaligners, #acceledent, #selfligatingbraces, and #propelorthodontics, whose hashtags were based in a pilot study made preliminary by the same researches to help identify a strategic time for gathering posts as well as to come up with the relevant keywords. Every researcher selected 10 posts of public profiles of orthodontists, five scientific-evidence-supported posts (SESP) and five without or with low scientific evidence (WLSE). All profiles included for analysis were freely accessible to the general public on the internet and the posts were available by June 2022. Next, the posts were discussed regarding scientific evidence, and a consensus among the three researchers was found regarding which posts should be included.

**Fig 1:**
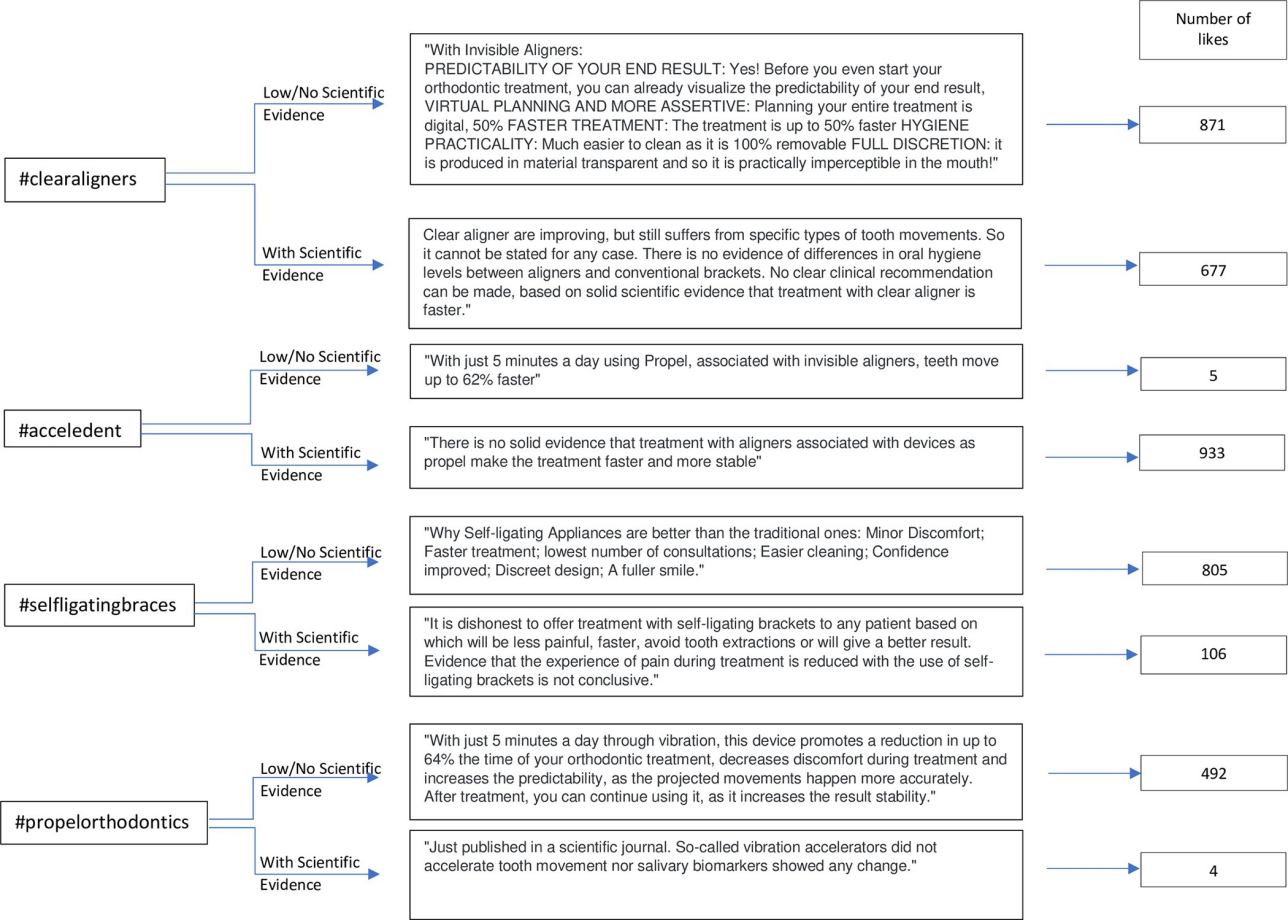
Texts for each hashtag SESP and WLSE.

Hence, four texts were used for scientific-evidence-supported posts and four without or with low scientific evidence—one SESP and WLSE for each hashtag. The chosen texts for each hashtag are presented in Fig 1. In addition, the original posts were uncharacterized, a generic image was used, the same image was repeated for the same hashtag, and the number of likes of each post was randomly assigned using < ramdomizer.org> between 0 and 1000. The username was “ortho.andrea”, proposing a neutral name to avoid any gender bias, with the same occurring with the name in the likes, which was called “Alisson Park”.

A self-administered questionnaire was developed by the authors according to [12, 13]. The questionnaire was divided into two parts. The first part presented four questions: “1. Do you usually read news and update yourself through social media? (A: Yes or No)”, “2. How do you search for medical/dental services for yourself? (A: internet [social media], family/friend referral, internet ad, I go anywhere [other])”, “3. Which social network do you use? (A: Instagram, TikTok, WhatsApp, Facebook, Twitter, LinkedIn, other)”, 4. “How much time do you spend connected to your social networks daily? (A: 30 minutes or less, more than 30 minutes to 1 hour, more than 1 hour to 2 hours, more than 2 hours to 3 hours, more than 3 hours)”.

The second part consisted of evaluating the posts created for the research. Eight images—four SESP and four WLSE—were evaluated through a visual analog scale. The posts were also randomized with the help of the website < ramdomizer.org> and were visible one at a time. After each post, the question “On a scale of 0 to 10, how much does this post convey the credibility of a post with real content with a scientific basis? (0 none– 10 a lot)” was applied. The questionnaires ended when the participant answered all four questions of the first part and eight questions of the second part.

### Questionnaire application, inclusion and exclusion criteria and participants’ backgrounds

For data collection, the questionnaire link was distributed by the researches via social media (Facebook, Instagram, LinkedIn, and WhatsApp) using the Qualtrics digital platform (Salt Lake City, UT, USA) and was made available from October 22^th^ of 2022 until November 16^th^ of 2022. Participants could access the questionnaire digitally using a computer, smartphone, or tablet. The questionnaire was available only in Portuguese and all Portuguese speakers who get the link could answer. The inclusion criteria were participants over 18 years old that completed all the questions in the questionnaire. Uncompleted surveys or that presented repeated number for all questions were excluded. Forty-nine participants were excluded and there were 385 participants included in the research.

### Reliability

For validation and reliability testing, the questionnaire was administered twice during the preliminary stage of data collection, with an interval of 20 days between the two administrations. For the first (test) and second (retest) administrations of the questionnaire, 30 subjects were included. The validation was based on previous studies [13–15]. Those subjects were not included in the final sample.

### Data analysis

The raw data was extracted from the Qualtrics software and using Microsoft Excel, the gathered data were collated and quantified in an electronic database (Microsoft, Inc., Redmond, WA, USA). The quantified data was exported to the SPSS software version 25 (IBM, Armonk, USA) in which was used for the statistical analysis. Cronbach’s alpha coefficient and paired Student`s T were used to measure internal consistency and confirm reliability. The mean and standard deviation of the numerical variables, as well as the total count and percentage of the qualitative variables, were calculated using descriptive statistics. To compare demographic data, paired student T test for sex and U-Mann-Whitney of ethnicity. To compare the dependent variables among groups, one-way ANOVA with Bonferroni correction due to multiple post-hoc tests and chi-square tests were used. A 5% (*p* < 0.05) level of significance was chosen.

The data collection and analysis method complied with the terms and conditions for the source of the data.

## Results

The sample of this study was composed of 175 laypeople (45.45%) (mean age 30.04 y, range 18–60), 58 dentistry students (15.06%) (mean age 21.79 y, range 18–41), 102 dentists (Non-orthodontists) (26.49%) (mean age 29.68 y, range 21–73), and 50 orthodontists (12.98%) (mean age 35.58 y, range 24–67. The general mean age for this study was 29.55 y, range 18–73, with 62 self-identifying as Black, 311 as White, and 12 as Hispanic. As for the difference regarding demographics and hashtags, statistical difference was found between sex (*p>0*.*05)* (Table 1), for the SESP #clearaligner *(p = 0*.*009*, male [mean 5.72 S ± 2.69], female [4.84 S ± 2.91]); and #selfligatingbraces (*p = 0*.*042* male [mean 6.09 ± 2.65], female [5.45 ± 2.75]) no significant difference was found when comparing with ethnicity. For time spend on social media, no difference was found when comparing sex and ethnicity.

**Table 1:** Socio-demographic characteristics of the participants.

Variable	Laypeople	Dentistry Student	Dentist non-Orthodontist	Orthodontist	Total	p-value
	n (%)	n (%)	n (%)	n (%)	n (%)	
**Number of participants**	175 (45.45)	58 (15.06)	102 (26.49)	50 (12.98)	385 (100)	.
**Age (mean ± SD)**	30.04 ± 10.13	21.79 ± 3.68	29.68 ± 9.20	35.58 ± 11.85	29.55 ± 10.24	.
**Sex**						**p < 0.05
Male	61 (35.85)	9 (15.41)	32 (31.37)	15 (30.00)	117 (30.38)	.
Female	114 (65.14)	49 (84.48)	70 (68.72)	35 (70.00)	268 (69.62)	.
**Ethnicity**						**p > 0.05
Black	42 (24.00)	4 (6.89)	12 (11.76)	4 (8.00)	62 (16.10)	.
White	126 (72.00)	52 (89.65)	87 (85.29)	46 (92.00)	311 (80.77)	.
Hispanic	7 (4.00)	2 (3.45)	3 (2.94)	-	12 (3.12)	.
**Do you usually read news and update yourself through social media?**						*p < 0.001
Yes	166 (94.85)	54 (93.10)	98 (96.07)	47 (94.00)	365 (94.80)	NA
No	9 (5.14)	4 (6.90)	4 (3.92)	3 (6.00)	20 (5.19)	NA

p value obtained from Chi-square test Statistical difference *p < 0.05

p value obtained from T-Student and U-Mann Whitney test Statistical difference **p < 0.05

NA–Not applied

**Fig 2:**
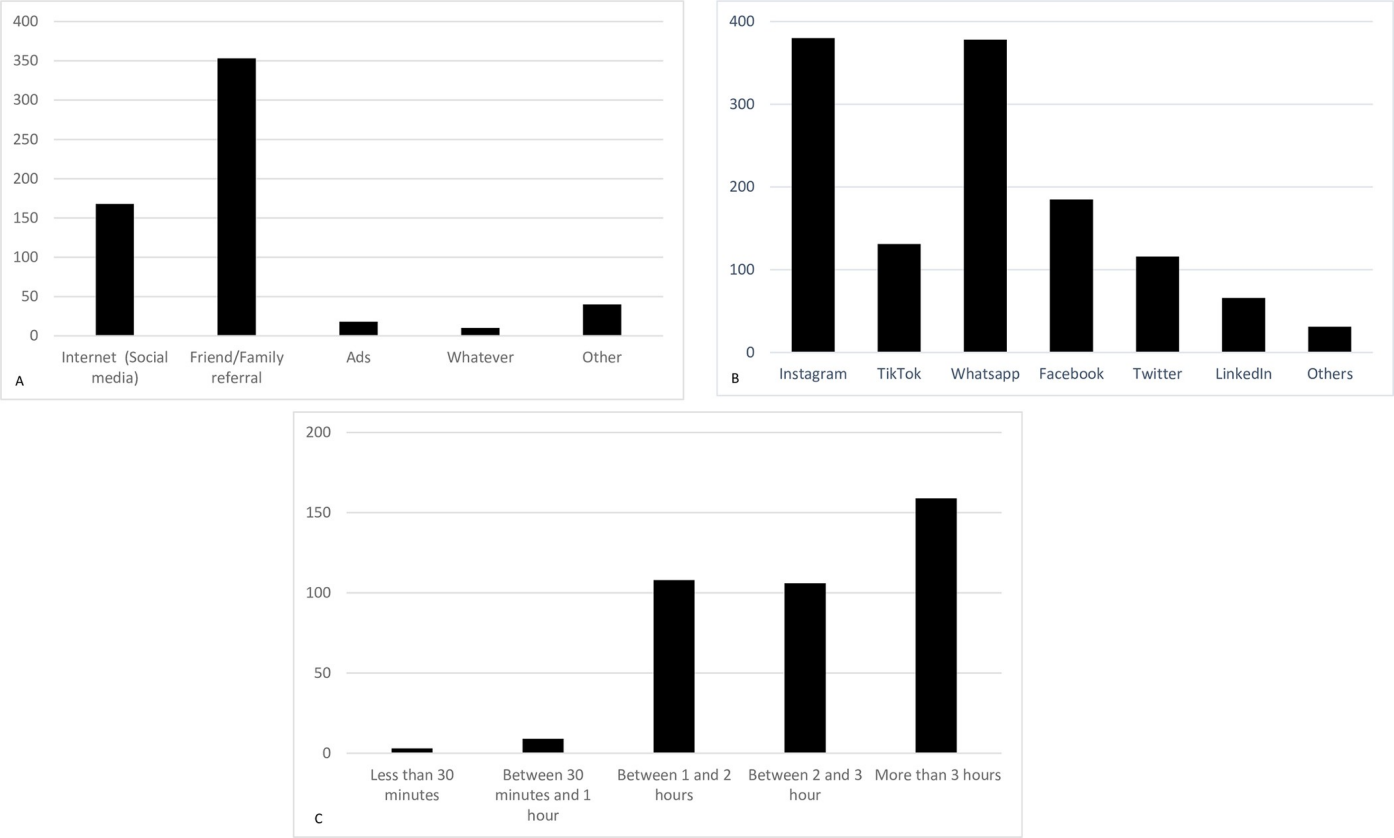
Charts regarding A. How do you search for medical/dental services for yourself?; B. Which social network do you use; C. How much time do you spend connected to your social networks daily?.

The questionnaire showed satisfactory internal consistency. Satisfactory agreement rates were also found between the test and retest results for all images categories evaluated (Cronbach’s alpha = 0.862, *p =* 0.673 for paired Student`s T test regarding reliability). The results showed that 365 (94.80%) participants use social media to read news and update themselves (Table 1). Regarding the question “How do you search for medical/dental services for yourself?”, the most chosen option was a referral through family and friends (n = 353; 91.68%), followed by the internet (social media) (n = 168; 43.63%) (Fig 2A). The most popular social media used by the participants of this research were Instagram (n = 380, 98.70% WhatsApp, (n = 378, 98.18%), Facebook (n = 185, 48.05%) and TikTok (n = 131, 34.02%), with 159 participants responding that they spend more than 3h on social media (n = 159, 41.29%), 106 stating spending between 2 and 3 h (n = 106, 27.53%), and 108 between 1 and 2h (n = 108, 28.05%). The distribution regarding preferred social media platforms and time spent on social media daily is available in Fig 2B and 2C, respectively.

Regarding posts on social media, for those with SESP, no statistical difference was seen when compared with the other groups for the Visual Analogue Scale (VAS). In the tests for the WLSE, statistical difference was perceived for the #selfligatingbraces (*p* < 0.001) and #propelorthodontics *(p =* 0.040*)* (Table 2). In the post-hoc tests, orthodontist’s group when compared with laypeople (*p* < 0.001) for the #selfligatingbraces, the group of orthodontists also showed a statistical difference for #propelorthodontics when compared with laypeople (*p* = 0.031) and dentists (*p* = 0.033). For the #clearaligners and #acceledent, no statistical difference was found (*p* > 0.05).

**Table 2:** ANOVA test between groups and posts and Bonferroni correction.

Hashtag	Groups	Mean	Standard Deviation	P value	Bonferroni Correction
#clearaligners Evidence supported	Laypeople	5.42	2.70	0.103	0.087
	Dentistry student	5.26	2.99		
	Dentist (non orthodontist)	4.55	2.92		
	Orthodontist	4.86	3.06		
#acceledent Evidence supported	Laypeople	5.08	2.85	0.170	0.171
	Dentistry student	4.94	2.94		
	Dentist (non orthodontist)	4.34	2.63		
	Orthodontist	5.28	2.83		
#selfligatingbraces Evidence supported	Laypeople	5.81	2.54	0.115	0.098
	Dentistry student	5.63	2.63		
	Dentist (non orthodontist)	5.07	2.97		
	Orthodontist	6.06	2.86		
#propelorthodontics Evidence supported	Laypeople	4.88	2.58	0.177	0.145
	Dentistry student	4.24	2.89		
	Dentist (non orthodontist)	4.43	2.49		
	Orthodontist	5.23	2.93		
#clearaligners NON Evidence supported	Laypeople	5.59	2.79	0.456	0.416
	Dentistry student	5.98	2.59		
	Dentist (non orthodontist)	5.29	2.79		
	Orthodontist	5.25	3.02		
#acceledent NON Evidence supported	Laypeople	4.64	2.90	0.254	0.209
	Dentistry student	4.13	2.45		
	Dentist (non orthodontist)	4.20	2.69		
	Orthodontist	3.76	2.49		
#selfligatingbraces NON Evidence supported	Laypeople^a^	5.64	2.70	p<0.001*	p<0.001*
	Dentistry student^ab^	4.81	2.57		
	Dentist (non orthodontist)^ab^	4.75	2.70		
	Orthodontist^b^	3.70	2.58		
#propelorthodontics NON Evidence supported	Laypeople^a^	4.95	2.75	0.040*	0.040*
	Dentistry student^a^	5.00	2.48		
	Dentist (non orthodontist)^a^	5.01	2.59		
	Orthodontist^b^	3.66	2.45		

385 participants

Different captions = Statistical Difference

Statistical difference when p<0.05*

## Discussion

Due to the growing number of social media users, mainly Instagram, and the search for information in these platforms, there has been a greater concern about the type of information that is transmitted by social media. Only two posts were identified in which orthodontists were able to perceive differences in relation to laypeople, in non-science-based posts. However, dental students and dentists (not orthodontists) did not show significant differences compared to laypeople.

Social media, particularly Instagram, has developed into an essential tool for patients and healthcare professionals in recent years [16]. The results in this study indicate that 97.04% of the population uses social media to update themselves and read the news, with Instagram as the social media preferred by the participants of this study. Of the participants, 43,00% responded that they search for orthodontics services on social media. Those results are similar to the findings reported in a study by Meira *et al*. [13], in which more than 95% of the participants used social media, particularly Facebook and Instagram, and nearly half of them used those platforms to look for medical care. This finding may justify the use of Instagram posts in this research and the importance of the present study, since the information passed through social networks can be considered not evidence-based [7, 17, 18].

Users generate and share information using social media as a method of electronic communication to engage in social networking. It is possible to share information, thoughts, and other content in form of videos, images, and texts. Over 3.5 billion people use social media every day, and it has been estimated that people spend an average of 3h each day online [1]. Regarding this study, most of the participants self-declared spending more than 3h on social media and the second-most declared spending between 2 and 3h online consuming information.

The Instagram platform has been highly popular in recent years, and many businesses and professionals have started to use it for promotions [19]. From the perspective of the patient, a dentist’s social media presence and a suitable interaction with them are key ways to connect with and attract new patients [20]. Hence, the role of social media for dentists and orthodontists has been increasing, but some concerning results have been shown in the literature regarding the quality of posts on various social media. Orthodontists should be aware of the content on social media platforms and educate and direct their patients toward correct and trusted Instagram resources [21]. Due the dubious quality of the posts, the present study sought to analyze whether there is a difference in the identification of posts with evidence and without evidence by orthodontists, laypersons, dentists and dental students. The results presented in this study are concerning, since orthodontists only differed for two hashtags out of the eight analyzed, this may suggest a lacking of scientific based evidence in orthodontic educational programs and also in general graduation programs. Most claims found in social media posts in one study [9] were not based on scientific evidence and were found to be false. Efforts should be made to promote the provision of accurate orthodontic information and to verify claims on social media platforms.

Regarding studies analyzing content in social media, a study using six hashtags (#*carrieremotion*, *#invisalign*, *#damonbraces*, *#acceledent*, *#propelorthodontics*, and *#myobrace*) 1730 posts were filtered to produce a sample of 300 Instagram posts. From those posts, less than 2% of the claims made were considered to be factually accurate [21]. Another study pointed out that orthodontists and maxillofacial surgeons should tell patients that the Instagram platform is not a reliable or acceptable source of information about orthognathic surgery because most of the posts are advertising or not based on scientific evidence [10]. Another study assessing clear aligner orthodontics-related YouTube videos offers an average quality, but overall information was low quality [11]. Similar results were found regarding the TikTok platform, where the content, reliability, and quality of videos found were poor [22]. One study showed that YouTube videos regarding orthodontic retainers could not be regarded as a sufficient source of knowledge about these appliances for patients [18]. It is possible even in orthodontics journals to find advertising that is not peer-reviewed and supported only for financial interest. Such content is mostly at a low level, with less than 2% of advertisements supported by high-level evidence [7].

It is claimed that specialists ought to direct patients to trustworthy information sources [11]. The overall need for scientific-based information on the internet is growing, and the variety of social media platforms in particular should be addressed by orthodontic experts because they can influence how patients behave [16]. The present study showed that poor-quality advertisements might influence not only patients but even dental professionals and orthodontists. Specifically, for the eight posts analyzed, only two showed observable statistical differences between general information (laypeople) and experts (orthodontists). Note that for the SESP, the descriptive data showed that orthodontists scored more points, and for the WLSE, orthodontists scored less for all subjects in the VAS, although it is concerning that orthodontists were able to identify only 25% of the posts differently from the general opinion. Moreover, dentists and dentistry students did not show any significant differences when compared to laypersons.

The only evidence-supported post where orthodontists had a lower VAS than other groups was about clear aligners. In this same subject, but in a non-evidence-supported post, the VAS value for orthodontists was the closest to the other groups. Furthermore, orthodontists rated WLSE posts with a higher VAS than SESP for #clearaligners. These findings suggest that orthodontists are inconsistent regarding the scientific evidence of clear aligners.

The posts in this study were created following some premises and general beliefs mostly caused by the high presence and marketing of companies on social media. Such claims include those clear aligners offers a faster treatment time, invisibility, and precise results predictable through software simulations. Current scientific evidence, however, shows these claims to be untrue [23–26]. The same pattern was followed for the posts about the self-ligating braces [27, 28] Propel and Acceledent [29–31].

A limitation of this study was not separating orthodontists into different degrees, such as experience, studies, and formation, although the general information presented in the posts should be taught in every serious dental school. The results must be evaluated carefully because our approach involved verifying the participants’ perceptions regarding Instagram posts. Perceptions of online content are highly heterogenetic with many variables, such as sexual dimorphism and possible demographic differences.

It is glaring that action should be taken regarding the potential hazards of social media, and patients must have access to credible web information. Given the limited reading and understanding of evidence-based methods among seasoned practitioners and more recent graduates, a downstream effect of incorrect information may also be felt among residents and general dentists. This study showed some concerning results regarding the knowledge of orthodontists regarding evidence-based practice and social media usage, which may be a correlation between time spent on social media and having the internet as a font of research and news. Changes in educational programs and how scientific science is consumed are required to enhance awareness of and reliance on evidence-based practices in orthodontics.

## Conclusion

Orthodontists were able to perceive differences in only two posts compared to the general perception (laypeople) regarding non- or low-scientific-evidence posts. Dental students and dentists (non-orthodontists) did not show any statistical differences when compared to laypeople.

Of the participants in the study, 97% used social media to keep informed and read the news on social media, and almost half searched for orthodontics services on social media. Instagram was the favorite social media platform.
